# Food perception without ingestion leads to metabolic changes and irreversible developmental arrest in *C. elegans*

**DOI:** 10.1186/s12915-018-0579-3

**Published:** 2018-10-08

**Authors:** Rebecca E. W. Kaplan, Amy K. Webster, Rojin Chitrakar, Joseph A. Dent, L. Ryan Baugh

**Affiliations:** 1 Department of Biology, Duke University, Box 90338, Durham, NC 27708-0338 USA; 2 Department of Biology, McGill University, Montreal, QC H3A 1B1 Canada

**Keywords:** Insulin, IGF, Perception, *C. elegans*, Diapause, L1 arrest, Food signal, Lipid metabolism

## Abstract

**Background:**

Developmental physiology is very sensitive to nutrient availability. For instance, in the nematode *Caenorhabditis elegans*, newly hatched L1-stage larvae require food to initiate postembryonic development. In addition, larvae arrested in the dauer diapause, a non-feeding state of developmental arrest that occurs during the L3 stage, initiate recovery when exposed to food. Despite the essential role of food in *C. elegans* development, the contribution of food perception versus ingestion on physiology has not been delineated.

**Results:**

We used a pharmacological approach to uncouple the effects of food (bacteria) perception and ingestion in *C. elegans*. Perception was not sufficient to promote postembryonic development in L1-stage larvae. However, L1 larvae exposed to food without ingestion failed to develop upon return to normal culture conditions, instead displaying an irreversible arrest phenotype. Inhibition of gene expression during perception rescued subsequent development, demonstrating that the response to perception without feeding is deleterious. Perception altered DAF-16/FOXO subcellular localization, reflecting activation of insulin/IGF signaling (IIS). The insulin-like peptide *daf-28* was specifically required, suggesting perception in chemosensory neurons, where it is expressed, regulates peptide synthesis and possibly secretion. However, genetic manipulation of IIS did not modify the irreversible arrest phenotype caused by food perception, revealing that wild-type function of the IIS pathway is not required to produce this phenotype and that other pathways affected by perception of food in the absence of its ingestion are likely to be involved. Gene expression and Nile red staining showed that food perception could alter lipid metabolism and storage. We found that starved larvae sense environmental polypeptides, with similar molecular and developmental effects as perception of bacteria. Environmental polypeptides also promoted recovery from dauer diapause, suggesting that perception of polypeptides plays an important role in the life history of free-living nematodes.

**Conclusions:**

We conclude that actual ingestion of food is required to initiate postembryonic development in *C. elegans*. We also conclude that polypeptides are perceived as a food-associated cue in this and likely other animals, initiating a signaling and gene regulatory cascade that alters metabolism in anticipation of feeding and development, but that this response is detrimental if feeding does not occur.

**Electronic supplementary material:**

The online version of this article (10.1186/s12915-018-0579-3) contains supplementary material, which is available to authorized users.

## Background

Perception of food affects metabolism and development in a variety of animals. Several observations suggest that sensory perception of food can regulate metabolism. For example, humans release insulin in response to the sight and smell of food [59]. Loss of olfactory neurons in mice reduces obesity and insulin resistance, and enhancing olfactory acuity does the reverse [55]. Blocking olfaction in *Drosophila* alters metabolism and extends lifespan; conversely, the longevity-extending effects of dietary restriction are partially reversed by exposure to food odors [41]. Likewise, in *Caenorhabditis elegans* sensory perception of a water-soluble “food signal” affects development of dauer larvae, a form of diapause as an alternative third larval stage [27], and perception of food also affects lifespan [1, 3, 33, 39]. However, the molecular cues that are sensed and their specific effects on organismal signaling and gene regulation are not well understood in any system.

*C. elegans* L1-stage larvae hatch in a state of developmental arrest (“L1 arrest” or “L1 diapause”) and require food to initiate development [8]. Insulin/IGF signaling (IIS) is a key regulator of L1 arrest [11, 26]. During starvation, *daf-16*/FOXO promotes L1 arrest by inhibiting development-promoting pathways [11, 35]. Feeding upregulates activity of the insulin-like peptides *daf-28*, *ins-6*, and *ins-4*, among others, which act as putative agonists for the only known insulin/IGF receptor *daf-2* [17]. *daf-2*/InsR signaling activates a conserved phosphoinositide 3-kinase (PI3K) cascade to antagonize DAF-16 by preventing its nuclear translocation, thereby promoting development [36, 42, 47, 49]. *daf-28*, *ins-6*, and *ins-4* are also critical to regulation of dauer development [18, 40], a form of developmental arrest that occurs in L3-stage larvae and is distinct from L1 arrest [8]. Together with their role in regulating L1 development, regulation of dauer development indicates that these insulin-like peptides are atop the organismal regulatory network governing postembryonic development. However, how these important insulin-like peptides are regulated in response to nutrient availability is unknown. IIS governs postembryonic development in insects as well [12, 15, 16, 53], suggesting that mechanisms mediating environmental influence on insulin-like peptide synthesis and secretion have broad significance.

Feeding in *C. elegans* is mediated by pumping of the neuromuscular organ called the pharynx [6]. The drug ivermectin paralyzes the pharynx by activating glutamate-gated chloride channels containing α-type channel subunits, increasing chloride conductance and inhibiting cellular depolarization [5, 19, 20, 63]. Several genes encoding glutamate-gated chloride channels in *C. elegans*, including *avr-14*, *avr-15*, and *glc-1*, confer sensitivity to ivermectin, but simultaneous mutation of all three of these genes produces substantial ivermectin resistance [21].

Here we used ivermectin to prevent feeding in *C. elegans* L1 larvae exposed to food. We show that perception of food without ingestion significantly alters gene expression and activates IIS but is not sufficient to initiate postembryonic development. To the contrary, perception without ingestion makes developmental arrest irreversible. We show that starved worms sense polypeptides in their environment as a food cue, likely in anticipation of feeding.

## Results

### Perception of food without ingestion renders developmental arrest irreversible

We used ivermectin to prevent feeding in order to uncouple the effects of food perception from ingestion. To limit effects of the drug outside the pharynx, we started with a highly ivermectin-resistant strain, the quadruple mutant *avr-14(vu47)*; *glc-3(ok321) avr-15(vu227) glc-1(pk54)* [21, 32], and rescued *avr-15* with a *myo-2* promoter for pharynx-specific expression. We made two versions of the strain with two different markers for analysis of L1 development: AJM-1::GFP to examine lateral epidermal seam cells and *Phlh-8*::GFP to examine the M-cell lineage. After making our primary observations with these transgenic strains in the quadruple mutant background, we used a wild-type background to facilitate genetic analysis of the phenotypes we discovered (see “Methods”).

**Fig. 1:**
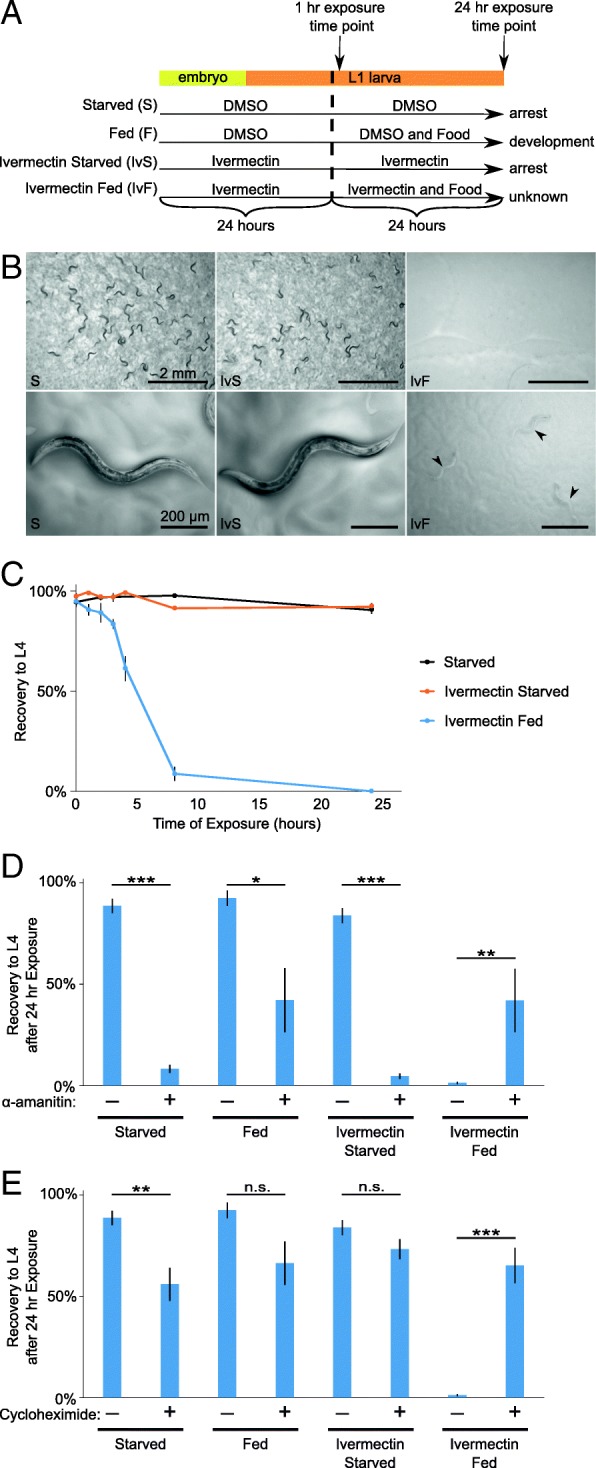
Prolonged exposure to food perception triggers an inability to recover that is mediated by a transcriptional/translational response. **a** Diagram of experimental setup with four standard treatment conditions over time. Dimethyl sulfoxide (DMSO; solvent). **b** Representative images of worm recovery after 3 days post starvation. Arrowheads indicate L1s. **c** L1 starvation recovery is plotted over time for three biological replicates. **d** The proportion of larvae that recovered to at least the L4 stage after 3 days of recovery is plotted for three to nine biological replicates. **e** The proportion of larvae that recovered to at least the L4 stage after 3 days of recovery is plotted for three to nine biological replicates. **b**–**e** ***p < 0.001, **p < 0.01; *p<0.05; unpaired *t* test. Error bars are SEM. Quadruple mutant transgenic background

Throughout this study, most experiments follow the basic setup seen in Fig. 1a. We prepared embryos by hypochlorite treatment of gravid adults and cultured them in either ivermectin or control (DMSO) conditions without food for 24 h where they hatch and enter L1 arrest. Various types of food or other substances were then added (vertical dashed line in Fig. 1a), and worms were typically analyzed 1 h or 24 h after this addition. To determine if ingestion was occurring, fluorescent beads were added to the cultures and worms were examined. Critically, fluorescent beads were not ingested in the ivermectin plus food (*E. coli* HB101) conditions (Additional file 1: Figure S1A). For initial characterization of the effects of food perception without ingestion, worms were exposed to experimental conditions for 24 h, then plated in standard laboratory conditions (on plates with *E. coli* OP50 but no ivermectin) and allowed to recover for 3 days. Worms exposed to ivermectin plus food failed to recover, remaining arrested in the L1 stage, while the controls recovered completely (Fig. 1b). That is, ivermectin treatment alone did not cause an irreversible arrest, but ivermectin plus food did. This striking phenotype was further characterized with a time series, revealing a near complete effect within about 8 h of exposure (Fig. 1c). Recovery to the L4 stage was chosen as an easy stage to reliably score. Though 24 h exposure generally rendered larvae capable of negligible if any growth, earlier time points associated with incomplete penetrance were associated with intermediate growth rates as well. Worms displayed significant failure to recover with as little as 1 mg/mL HB101 during ivermectin treatment (Additional file 1: Figure S1B), and worms were at least as sensitive to *E. coli* OP50 and HT115 (Additional file 1: Figure S1C). These results indicate that the observed effect of food perception is not limited to a particular strain or high density of *E. coli*. A robust irreversible arrest phenotype following exposure to food in the presence of ivermectin was also observed in wild-type worms (see below; Fig. 3i), indicating that it is not an artifact of the quadruple mutant transgenic strain. Together, these results reveal a potent effect of exposure to *E. coli* without feeding on the ability of *C. elegans* larvae to recover from starvation-induced developmental arrest.

Feeding causes a significant change in transcription and translation in *C. elegans* L1 larvae [9, 44, 60]. We hypothesized that food perception evokes a gene expression response that is deleterious without feeding, explaining the inability of larvae exposed to food and ivermectin to subsequently resume development. Worms were treated with the drug α-amanitin, which inhibits transcription [45, 57, 65], slightly before and during food exposure. Inhibiting transcription significantly increased recovery in worms exposed to ivermectin and food (Fig. 1d). As a complementary approach, worms were treated with cycloheximide to block translation [45] in a similar manner. This treatment also significantly improved recovery in worms exposed to ivermectin and food (Fig. 1e). Notably, inhibition of expression by either method decreased recovery in conditions where perception was consistent with feeding state (i.e., starved, ivermectin starved, and fed), as if gene expression was appropriate in such conditions, supporting fitness. Strikingly, inhibiting expression actually increased recovery in the condition in which perception is inconsistent with feeding state (ivermectin fed), as if gene expression is inappropriate to the starved state, compromising fitness. Together, these results suggest that food perception alters gene expression and that this change in expression affects the animal adversely if it is not accompanied by feeding.

### Food perception evokes a gene expression response similar to feeding

**Fig. 2:**
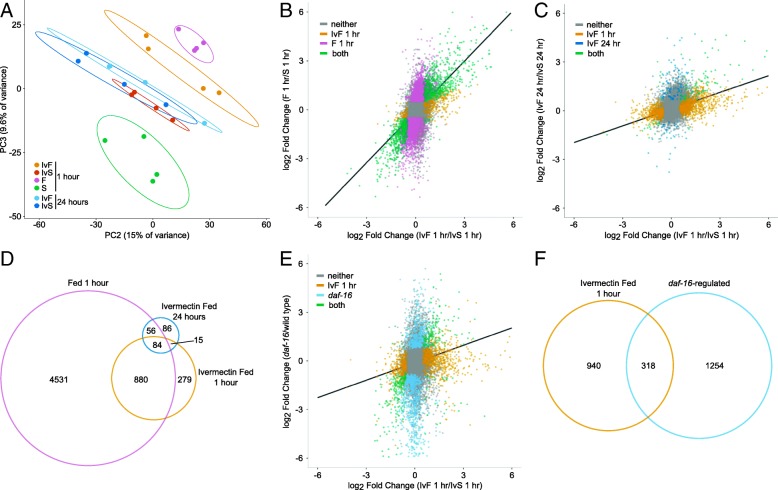
mRNA-seq reveals transcriptional effects of food perception. **a** PCA of four biological replicates is plotted. Ellipses represent 80% confidence intervals, the probability for two of which not overlapping by chance is approximately 0.04. **b**, **c** Mean gene expression changes of four biological replicates are plotted for the indicated conditions. **d** Overlap between genes significantly affected by food at 1 h, ivermectin and food at 1 h, and ivermectin and food at 24 h is plotted. **e** Mean gene expression changes of two to four biological replicates is plotted for the indicated conditions and genotypes. The universal set of genes considered includes only those analyzed in both studies. **f** Overlap between genes significantly affected by ivermectin and food with genes significantly affected by *daf-16* is plotted with the same universal set as in **e**. **a**–**f** Quadruple mutant transgenic background, except for *daf-16* in wild-type background

We performed mRNA-seq to characterize the effects of food perception on gene expression. We assayed quadruple mutant transgenic larvae that were exposed to ivermectin and food for 1 h or 24 h to distinguish relatively immediate and long-term effects. We assayed larvae exposed to ivermectin without food at the same time points for reference, as well as larvae that were fed or starved without ivermectin for 1 h (a 24-h time point was not included since the fed larvae would have developed to the L3 stage). Principal component analysis revealed a large effect of ivermectin, with ivermectin treatment correlating with the first component (Additional file 2: Figure S2). Feeding significantly affected mRNA expression, as expected, and the second and third principal components separated the fed and starved worms (Fig. 2a). Notably, worms exposed to ivermectin and food for 1 h were different from worms starved with ivermectin, instead showing greater similarity to fed worms. However, by 24 h of exposure to ivermectin and food, the expression profile was not significantly different from its starved control. Likewise, 1258 genes were differentially expressed at 1 h comparing ivermectin with food to ivermectin starved, but only 241 genes were differentially expressed in the same comparison at 24 h (false discovery rate (FDR) < 0.05 and an absolute log_2_ fold change of greater than 0.5; Additional file 3: Dataset S1). These results show that perception of food alters gene expression initially but that this effect subsides over time.

We wondered how well correlated the gene expression response to food perception is with feeding. The magnitude of the feeding response was larger, with 5551 differentially expressed genes at 1 h compared to 1258 genes in the presence of ivermectin. These gene expression changes were very well correlated, with 98.8% of genes differentially expressed in both conditions changing in the same direction (Fig. 2b). Indeed, the vast majority of genes affected by food with ivermectin were also affected by feeding (Fig. 2d, hypergeometric *p* value = 6.8e−353). These results indicate that perception of food evokes a similar, though reduced, gene expression response to feeding. The response to food in the presence of ivermectin at 1 h and 24 h was also well correlated, with 91.9% of genes differentially expressed at both times responding in the same direction (Fig. 2c). Indeed, there was significant overlap in the differentially expressed genes at both time points (Fig. 2d, hypergeometric *p* value = 3.7e−60). These results support the conclusion that perception of food initially alters gene expression in a way that resembles the feeding response but that this response to perception diminishes over time.

As an effector of IIS, *daf-16*/FOXO is an important regulator of gene expression during L1 starvation [31, 35]. Since DAF-16 is inactivated by IIS in response to feeding, we hypothesized that it is also inactivated by perception of food, contributing to the resulting gene expression response. We previously identified 1572 genes differentially expressed in a *daf-16* null mutant compared to wild-type during L1 starvation [35]. These differences in gene expression correlated with the effect of food in the presence of ivermectin at 1 h, with 88.4% of the genes significantly affected in both comparisons responding in the same direction (Fig. 2e). There was also significant overlap in the genes affected in both comparisons (Fig. 2f, hypergeometric *p* value = 2.5e−98). These results suggest that perception of food in starved *C. elegans* larvae reduces *daf-16*/FOXO activity.

### Perception of food activates insulin/IGF signaling

**Fig. 3:**
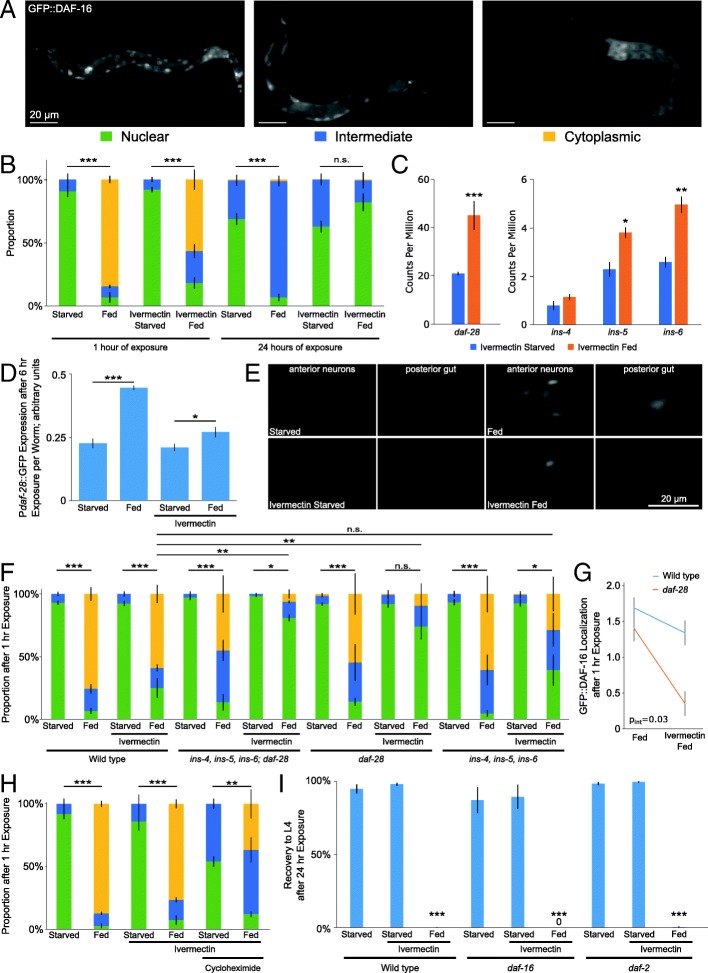
GFP::DAF-16 localization response to food perception is insulin-dependent. **a** Representative images of how GFP::DAF-16 localization was characterized. **b** GFP::DAF-16 localization is plotted for three biological replicates. **c** Average transcript abundance from four biological replicates of mRNA-seq is plotted for selected insulin-like peptides. Nominal *p* values displayed. **d** Averages of P*daf-28*::GFP fluorescence intensity normalized by optical extinction per worm using the COPAS BioSorter are plotted for four biological replicates. Exposure to HB101 was 6 h. **e** Representative images of P*daf-28*::GFP transcriptional reporter gene are presented. **f** GFP::DAF-16 localization is plotted for three to six biological replicates. **g** GFP::DAF-16 localization is plotted for three to six biological replicates. The two-way ANOVA interaction *p* value is listed. **h** GFP::DAF-16 localization is plotted for three biological replicates. **i** The proportion of larvae that recovered to at least the L4 stage after 3 days of recovery is plotted for three to four biological replicates. **b**–**i** ***p < 0.001, **p < 0.01; *p<0.05; unpaired *t* test. Error bars are SEM, except for in **d** where they are standard deviation. **a**, **b**, **d**–**i** Wild-type background. **c** Quadruple mutant transgenic background

Similarity in the gene expression responses of quadruple mutant transgenic worms exposed to food in the presence of ivermectin (compared to no food with ivermectin) and a starved *daf-16*/FOXO mutant (compared to starved wild-type worms) suggests that perception of food reduces DAF-16 activity. We further hypothesized that such a decrease in activity is due to activation of IIS. Since IIS regulates subcellular localization of DAF-16 [30, 64], perception of food should affect localization if this hypothesis is correct. We analyzed GFP::DAF-16 localization in a wild-type background, rather than in the quadruple mutant transgenic strain (see “Methods”), because the fluorescent markers in the quadruple mutant transgenic strain would have interfered with observation of GFP::DAF-16. We categorized GFP::DAF-16 localization as nuclear, intermediate, or cytoplasmic (Fig. 3a). As expected [30], GFP::DAF-16 was primarily nuclear during starvation and primarily cytoplasmic after 1 h exposure to food (Fig. 3b). One hour exposure to food with ivermectin also significantly shifted GFP::DAF-16 to the cytoplasm, supporting our hypothesis that perception of food activates IIS. Also as expected [64], GFP::DAF-16 was relatively less nuclear after 24 h of starvation than at 1 h. However, after 24 h exposure to food, there was no difference between ivermectin-fed and ivermectin-starved worms, in contrast to 1 h exposure to food in the presence of ivermectin. Similar to mRNA-seq results showing a substantially larger effect at 1 h exposure to food in the presence of ivermectin than at 24 h (Fig. 2b–d), this result suggests that perception of food is sufficient to alter DAF-16 localization initially but not to maintain it in the cytoplasm. GFP::DAF-16 localization also responds to other bacterial strains in the presence of ivermectin (Additional file 4: Figure S3A). These results suggest that perception of each of the bacterial strains typically used as food in the lab can activate IIS.

The *C. elegans* genome encodes 40 insulin-like peptides, and genetic analysis suggests they function as agonists or antagonists of DAF-2/InsR [51]. We wondered if the expression of specific insulin-like peptides was affected by food perception, potentially accounting for activation of IIS. Based on our mRNA-seq results, expression of two insulin-like peptides, *ins-12* and *ins-24*, was significantly downregulated after 1 h exposure to food in the presence of ivermectin (log_2_FC = − 2.3, FDR = 0.004 and log_2_FC = − 0.72, FDR = 0.001, respectively). Previous characterization revealed complex expression of *ins-12* in fed and starved L1 larvae as well as opposite functional effects on dauer entry and exit, precluding classification as a putative agonist or antagonist [17, 24]. *ins-24* expression decreases in response to feeding during recovery from L1 arrest, suggesting it functions as an antagonist, though this conclusion is not supported by functional analysis [17]. Nonetheless, downregulation of *ins-12* and *ins-24* in response to food perception tentatively suggests these two insulin-like peptides may function as DAF-2 antagonists in this context.

In contrast to putative DAF-2/InsR antagonists, putative agonists have been functionally characterized during L1 arrest and recovery. The insulin-like peptides *daf-28*, *ins-4*, *ins-5* and *ins-6* are transcriptionally upregulated by feeding L1 larvae, and *daf-28*, *ins-4* and *ins-6* promote L1 development [17], consistent with function as DAF-2/InsR agonists in regulation of dauer development [18, 40, 51]. We found that *daf-28*, *ins-5*, and *ins-6* transcripts were significantly upregulated after 1 h exposure to food in the presence of ivermectin (Fig. 3c). We used the COPAS BioSorter to quantify whole-worm fluorescence of a P*daf-28*::GFP transcriptional reporter, supporting the conclusion that *daf-28* transcription increases in response to food perception (Fig. 3d). This reporter was expressed in anterior neurons, previously identified as the amphid chemosensory neurons ASIL/R and ASJL/R [40], and the posterior intestine with higher expression after 6 h feeding (Fig. 3e), as expected [17]. Consistent with the COPAS results and mRNA-seq, the reporter was also brighter after exposure to food in the presence of ivermectin. These results reveal transcriptional upregulation of putative DAF-2/InsR agonists including *daf-28* as an initial response to perception of food, consistent with activation of IIS.

We performed genetic analysis of our candidate DAF-2/InsR agonists to determine functional relevance in activation of IIS in response to food perception. We used null alleles for the insulin genes and assayed GFP::DAF-16 localization in an otherwise wild-type background. As a control, mutation of *daf-2*/InsR completely blocked the effects of food on GFP::DAF-16 localization (Additional file 4: Figure S3B). Functional redundancy among the 40 insulin genes is common [51], particularly in regulation of L1 arrest and recovery, but simultaneous disruption of multiple genes can reveal loss-of-function phenotypes [17]. *ins-4*, *5* and *6* are clustered on chromosome II, and a deletion allele that removes all three causes constitutive dauer formation [34]. Combination of this deletion allele with a *daf-28* deletion allele to simultaneously disrupt all four putative agonists reduces L1 starvation survival [17]. This compound mutant retained the response to feeding, with GFP::DAF-16 moving from the nucleus to the cytoplasm (Fig. 3f), revealing robust regulation of DAF-16 localization. However, the change in localization in response to food in the presence of ivermectin was significantly reduced in the compound mutant (Fig. 3f). The *daf-28* deletion alone mimicked the behavior of the compound mutant, but the *ins-4*, *5*, *6* deletion alone did not, suggesting *daf-28* specifically mediates the response to food perception. To examine this closer, we plotted the data for wild-type and the *daf-28* mutant separately, focusing on the effect of food in the presence and absence of ivermectin (Fig. 3g). These data show a specific effect of *daf-28* on the response to food in the presence of ivermectin (two-way ANOVA *p* values for interaction between genotype and presence or absence of ivermectin: *daf-28* = 0.03, *ins-4*, *5*, *6*; *daf-28* = 0.02, *ins-4*, *5*, *6* = 0.29). These data suggest that *daf-28* plays a critical role in mediating the initial response to food perception on IIS activity, and they suggest that overlapping function of insulin-like peptides provides a more robust response to feeding than perception alone.

We reasoned that perception of food likely promotes secretion, in addition to synthesis, of DAF-28 and other putative DAF-2 agonists from chemosensory neurons, providing a rapid response to environmental conditions. To address this hypothesis, we treated worms with cycloheximide to block translation and examined GFP::DAF-16 localization. Even in the presence of cycloheximide, exposure of ivermectin-treated larvae to food caused a cytoplasmic shift in GFP::DAF-16 localization (Fig. 3h). Cycloheximide treatment constitutes a massive perturbation, affecting innumerable processes, but this result is consistent with perception of food affecting insulin-like peptides posttranslationally, possibly at the level of secretion. However, the shift in GFP::DAF-16 localization was incomplete with cycloheximide treatment, suggesting incomplete inhibition of translation and/or additional regulatory mechanisms. Together with our results showing an effect of food perception on *daf-28* transcription (Fig. 3c–e), these results suggest that food perception affects insulin-like peptide activity at multiple levels of regulation, in particular transcriptionally and posttranslationally.

Given the effects of food perception on IIS, we hypothesized that IIS mutants affect the irreversible arrest resulting from perception without feeding. If cytoplasmic localization of DAF-16 during starvation were sufficient to cause the irreversible arrest phenotype, then *daf-16* mutants should not be able to recover following starvation. *Daf-16* mutants are starvation-sensitive, but they nonetheless can be starved and retain the ability to recover upon feeding (Fig. 3i). Testing for necessity of IIS activation in causation of the irreversible arrest phenotype would require blocking IIS during food exposure. Null *daf-2*/InsR mutations are inviable [50], so we can only partially block IIS with a *daf-2* partial loss-of-function mutant. The *daf-2*/InsR mutant did not display increased recovery after exposure to food in the presence of ivermectin (Fig. 3i). Together, these results suggest that fully functional IIS is not necessary nor is altered IIS sufficient to cause the irreversible arrest. It is possible that altered IIS is irrelevant to the irreversible arrest phenotype, but we believe instead that the basis for the phenotype is complex and caused by alteration of multiple pathways such that manipulation of any one pathway alone during starvation will not cause irreversible arrest.

### Perception of food is not sufficient to promote development

**Fig. 4:**
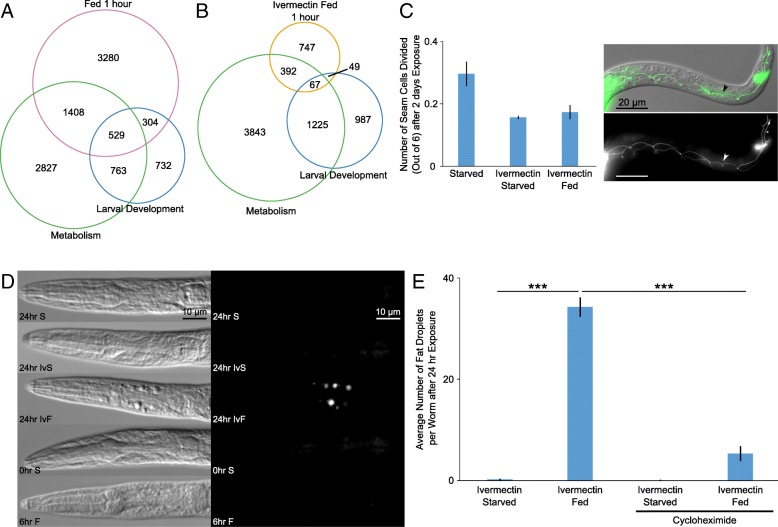
Food perception significantly affects metabolism but not development. **a** Overlap between genes significantly affected by food with genes in the metabolic process and larval developmental process GO terms is plotted. **b** Overlap between genes significantly affected by ivermectin and food with genes in the metabolic process and larval developmental process GO terms is plotted. **c** The average number of seam cell divisions, out of six possible, is plotted for three biological replicates. Scoring was done 2 days after HB101 addition. **d** Representative DIC and GFP channel images of fixed worms following Nile red staining are presented. **e** Quantification of fat droplets in Nile red staining is plotted for three to four biological replicates. **c**–**e** ***p < 0.001, **p < 0.01; unpaired *t* test. Error bars are SEM. **a**–**c** Quadruple mutant transgenic background. **d**, **e** Wild-type background

We used Gene Ontology (GO) term enrichment analysis of our mRNA-seq results to get a broad view of the processes affected by perception of food. The response to feeding for 1 h revealed significant overlap with metabolism genes (hypergeometric *p* value = 8.6e−92) and larval development genes (Fig. 4a, hypergeometric *p* value = 9.8e−39). The response to food exposure for 1 h in the presence of ivermectin also revealed significant overlap with metabolism genes (Fig. 4b, hypergeometric *p* value = 5.0e−23) but not with larval development genes (hypergeometric *p* value = 0.89). Furthermore, genes differentially expressed in response to both food exposure in the presence of ivermectin and feeding were enriched for metabolic GO terms, while genes differentially expressed in response to feeding but not exposure to food in the presence of ivermectin were enriched for a variety of GO terms related to development (Additional file 3: Dataset S1, Additional file 5: Tables S1 and S2). These results suggest that perception of food affects metabolism but not development.

The lateral epidermal seam cells are the first cells to divide in developing L1 larvae [61], and they divide very rarely during L1 arrest [11, 35]. We used an AJM-1::GFP reporter for adherens junctions to visualize seam cell membranes and count divisions of the cells v1–6 [29]. Consistent with the results of GO term analysis, exposure to food for 2 days in the presence of ivermectin did not cause seam cell divisions (Fig. 4c). There were also no M-cell divisions (data not shown). These results with the most stringent assay available indicate that perception of food is not sufficient to promote detectable postembryonic development.

### Perception of food alters lipid metabolism

We were particularly interested in distinct effects of food perception alone compared to feeding, since exposure of larvae to food in the presence of ivermectin caused irreversible developmental arrest. We therefore examined GO term enrichments among genes significantly affected by food exposure in the presence of ivermectin but not by feeding after 1 h (Table 1). This set of genes was enriched for fatty acid and lipid metabolic GO terms, especially those related to catabolism and oxidation. Examination of individual genes contributing to these GO term enrichments identified genes involved in fatty acid breakdown (e.g., *cpt-1,2*, *acox-1,2*, *acdh-12*, *ech-1.1,1.2*, and *acaa-2*) as upregulated after 1 h of food perception. However, genes involved in fatty acid biogenesis (e.g., *fasn-1* and *acs-5*) were also upregulated, suggesting complex effects on lipid metabolism.

**Table 1:** Food perception affects lipid metabolism-related GO terms

GO term description	FDR	Enrichment	Number of genes in GO term	Number of genes in target set	Number of genes in overlap
Fatty acid beta-oxidation	8.93E−08	16.69	36	227	10
Lipid oxidation	2.62E−07	15.02	40	227	10
Fatty acid oxidation	2.46E−07	15.02	40	227	10
Fatty acid catabolic process	1.95E−08	14.71	49	227	12
Monocarboxylic acid catabolic process	2.71E−08	14.13	51	227	12
Fatty acid metabolic process	1.97E−11	11.30	101	227	19
Cellular lipid catabolic process	4.34E−07	10.30	70	227	12
Carboxylic acid catabolic process	5.10E−08	10.01	84	227	14
Organic acid catabolic process	5.67E−08	10.01	84	227	14

Top GO terms ranked by enrichment values (at least 10-fold) are listed for genes significant in IvF vs. IvS at 1 h but not F vs. S at 1 h from four biological replicates of mRNA-seq. A quadruple mutant transgenic strain was used for mRNA-seq. Full list is available in Additional file 3: Dataset S1

Microscopic analysis of larvae further supports the conclusion from GO term enrichments that lipid metabolism is affected by perception of food. Differential interference contrast microscopy revealed numerous, prominent droplets throughout the body and around the pharynx, as if in the body cavity, after prolonged exposure to food in the presence of ivermectin (Fig. 4d). Given their appearance and GO term enrichments (Table 1), we hypothesized that these are lipid droplets. Nile red staining of fixed L1 larvae confirmed that these droplets contained lipid, supporting our hypothesis (Fig. 4d). Staining was done in a wild-type background, as opposed to a quadruple mutant transgenic background, to avoid interference from fluorescent reporters. Starved L1 larvae, either shortly after hatching or 24 h later, did not contain such lipid droplets. Fed L1 larvae developed small lipid droplets in the intestine, while the droplets in worms exposed to food and ivermectin for 24 h were more varied in size and location. Cycloheximide treatment significantly reduced the number of lipid droplets caused by food in the presence of ivermectin (Fig. 4e). This result suggests that the change in lipid metabolism that gives rise to these abnormal lipid droplets is due to differential gene expression, consistent with our mRNA-seq results (Table 1). We conclude that the gene expression response to food perception alters lipid metabolism, resulting in abnormal accumulation of large lipid droplets in the body cavity.

### Polypeptides serve as an environmental cue for food

**Fig. 5:**
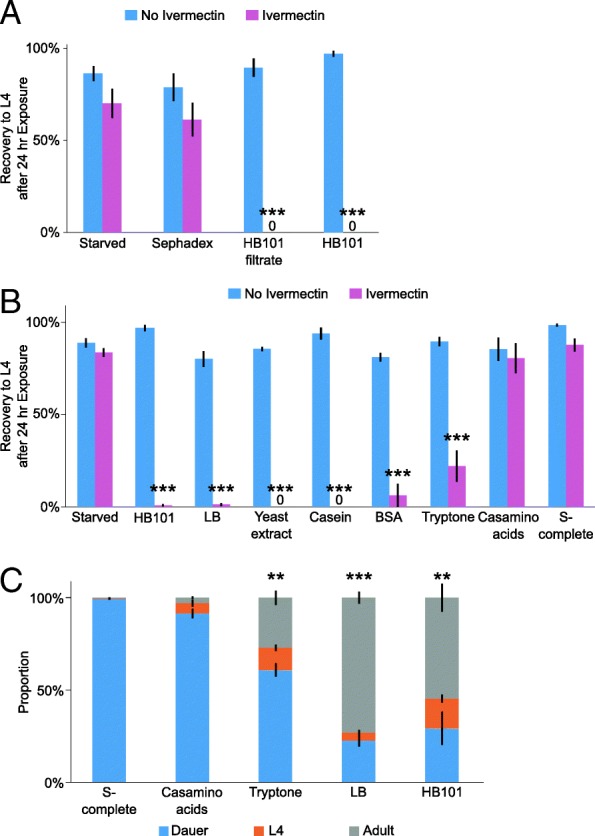
Perception of food cues affects L1 and dauer recovery. **a**–**b** The proportion of larvae that recovered to at least the L4 stage after 3 days of recovery is plotted for three to 13 biological replicates. **c** Recovery from dauer after 3 days in each condition is plotted for three to four biological replicates. **a**–**c** ***p < 0.001, **p < 0.01, *p < 0.05; unpaired *t* test. Error bars are SEM. **a**, **b** Quadruple mutant transgenic background. **c** Wild-type background

Nematodes rely on mechanosensory and chemosensory cues to regulate locomotion, development, pathogen avoidance, feeding, and mating [7, 28]. *C. elegans* respond to mechanosensory stimulation when encountering a bacterial lawn, which can be mimicked with Sephadex beads [58]. To test whether the effects of food perception were due to mechanosensation or chemosensation, we assayed the ability to recover after starvation in the presence of ivermectin and Sephadex beads or HB101 bacterial filtrate, respectively. We found that Sephadex beads did not affect starvation recovery, while HB101 filtrate prevented recovery as strongly as HB101 itself (Fig. 5a, Additional file 1: Figure S1A). These data suggest that the deleterious effect of food perception without ingestion is via chemosensation and not mechanosensation.

Since the relevant modality of perception appeared to be chemosensory, we wanted to identify a molecular component of bacterial food that functions as an environmental cue for the worm. We found that LB medium, a common nutrient broth for culturing *E. coli*, as well as its components, yeast extract and tryptone, caused irreversible arrest in worms exposed to them in the presence of ivermectin, similar to the effect of HB101 (Fig. 5b). Yeast extract results from autolysis of *S. cerevisiae* and contains a complicated mixture of amino acids, nucleic acids, peptides, carbohydrates, and vitamins. Tryptone is a tryptic digest of the protein casein, resulting in peptides of varying lengths (dipeptides and larger). Since tryptone is much simpler than yeast extract, we decided to focus our investigation there. We tested undigested casein and casamino acids, which is casein that has been through acid hydrolysis to produce free amino acids. We also tested bovine serum albumin (BSA) as another form of protein. We found that exposure to casein or BSA in the presence of ivermectin caused the irreversible arrest phenotype while casamino acids did not (Fig. 5b). Since casamino acids do not contain polypeptide, together these results suggest that polypeptide is perceived. We also exposed ivermectin-treated larvae to a solution of the ten essential amino acids for *C. elegans*, ethanol, glucose, and a combination of all three and found that none of these significantly affected recovery (Additional file 6: Figure S4A). Perception of polypeptides and other potential food cues also caused GFP::DAF-16 to translocate to the cytoplasm (Additional file 6: Figure S4B, C), consistent with activation of IIS. When otherwise starved larvae were permitted to ingest polypeptide or other potential cues, they supported survival (Additional file 6: Figure S4D) but not development (based on the M-cell division assay; data not shown), as if providing an incomplete source of nutrition. This treatment also compromised the ability of larvae to subsequently recover in standard culture conditions (Additional file 6: Figure S4E, F). This effect is reminiscent of the effect of exposure to food in the presence of ivermectin, consistent with a deleterious effect of perception of food-associated cues without being fed a complete source of nutrition. In summary, we conclude that starved worms perceive polypeptides, presumably as a food-associated cue, though other cues are likely to also be involved.

We wanted an alternative and arguably more ecologically relevant approach than using ivermectin to determine if starved worms perceive polypeptide. Dauer larvae have an internal plug blocking the pharynx and do not pump, preventing them from feeding [14, 54]. While both L1 arrest and dauer arrest are naturally occurring, dauer larvae are naturally incapable of feeding, but blocking ingestion in L1 larvae has to be experimentally induced (e.g., with ivermectin). Dauer larvae therefore provide a compelling alternative to ivermectin treatment for the analysis of food perception without ingestion. We found that tryptone and LB promoted dauer recovery, as did HB101, while casamino acids and the buffer S-complete did not (Fig. 5c). These results further support the conclusion that *C. elegans* perceive environmental polypeptides when starved as a food-associated cue.

## Discussion

We sought to uncouple the effects of food perception and ingestion on development, gene expression and metabolism of the nematode *C. elegans*. We report that perception is not sufficient to promote postembryonic development, but that it activates IIS and alters gene expression and lipid metabolism, resulting in an irreversible arrest phenotype. We also report that starved larvae sense environmental polypeptides, as if worms use them as a food-associated cue to anticipate feeding and development.

The most striking phenotype we report is the irreversible developmental arrest of larvae that are starved in the presence of food, so that they perceive food without eating it. Notably, ivermectin binding has been characterized as irreversible [19, 32, 63]. These studies involved very different time scales from ours, and they used ivermectin doses 50–100-fold greater than us. Nonetheless, we considered irreversible binding as an explanation for irreversible arrest, but several lines of evidence suggest otherwise. Worms exposed to the relatively low dose of ivermectin we used without food almost completely recover. Also, recovery was rescued by blocking transcription or translation during exposure to ivermectin and food. In addition, we see a similar reduction in recovery rate in otherwise starved L1 larvae exposed to food cues. This observation along with the effect of ivermectin and food suggests that perception of food cues without ingestion of complete nutrition underlies the irreversible arrest phenotype. We found that food perception activates IIS, affecting gene expression, but genetic analysis of a *daf-16* null mutant suggests that activation of IIS during starvation does not on its own cause an irreversible arrest phenotype (Fig. 3i). Furthermore, irreversible arrest is still triggered by food in ivermectin-treated animals in the absence of a fully functional IIS pathway, as revealed by analysis of *daf-2(e1370)*, a partial-loss-of-function allele. We interpret this as an indication that food perception alters multiple pathways, which may redundantly cause the phenotype, but that modulation of any one of them is not sufficient to cause irreversible arrest. We speculate that perception of food alters metabolism to prime the animal for feeding and development, but that these physiological changes lead to the loss of the stress-resistant properties that normally accompany L1 arrest such that they are detrimental if not accompanied by feeding.

We present evidence that food perception elicits a gene expression response that is largely subsumed by the feeding response and that this response is in part due to activation of IIS. Reduction of IIS and activation of DAF-16/FOXO during L1 starvation affects metabolic gene expression, promoting carbon flux through the glyoxylate shunt, gluconeogenesis, and into the disaccharide trehalose [31]. The gene expression response to feeding for 1 h tentatively suggests reversal of this starvation response, including apparent downregulation of the glyoxylate shunt (*icl-1* expression) and trehalose synthesis (*tps-1* and *tps-2* expression). Of these gene expression changes, downregulation of the trehalose synthase gene *tps-1* alone was also observed in response to food perception for 1 h. While this is consistent with a decrease in trehalose synthesis in response to food perception, it is a relatively limited effect on central carbon metabolic gene expression, and we have no additional evidence that carbohydrate metabolism is affected by food perception.

Insulin-like peptides *daf-28*, *ins-6* and *ins-4* govern postembryonic development, and their transcription is positively regulated by nutrient availability [17, 18, 40]. We show that *daf-28* transcription is upregulated by perception of food and that it plays a specific role in activating IIS in response to perception. That is, *daf-28* was specifically required for food perception to cause GFP::DAF-16 translocation to the cytoplasm, though it was dispensable for translocation in response to feeding, suggesting overlapping function with other insulin-like peptides in the latter but not former case. GFP::DAF-16 translocation to the cytoplasm in response to food perception occurred in the presence of the translational inhibitor cycloheximide, consistent with perception affecting insulin-like peptide activity posttranslationally, possibly at the level of secretion.

The gene expression response and activation of IIS were relatively transient, as if larvae initially respond to food perception but this response is not maintained without feeding and ingestion of nutrients. We imagine that the transient nature of this response is due to habituation of perception or antagonism from internal starvation signals, or a combination of the two. Upregulation of IIS during L1 starvation promotes cell division [17], but perception of food did not, though IIS was activated. We believe the transient nature of IIS activation by food perception explains the lack of postembryonic development. Despite the transient nature of the responses to food perception, they nonetheless have physiological consequences as demonstrated by accumulation of lipid droplets and irreversibility of developmental arrest.

Where the gene expression response to food perception differed from the feeding response, GO term enrichments suggest an effect on lipid metabolism. Food perception specifically increased expression of genes related to both catabolism and synthesis of fatty acids. Expression analysis of adults treated with ivermectin to limit food consumption also suggested an effect on lipid catabolism gene expression [37]. Simultaneous increase of catabolism and synthesis is paradoxical, but we imagine this could reflect interconversion of lipid species and/or result from measuring mRNA expression in whole animals, with metabolism varying among tissues. Nonetheless, perception of food caused accumulation of abnormal, prominent lipid droplets in the body cavity, consistent with an effect on lipid metabolism and storage.

Perception of a “food signal” from bacterial cultures or yeast extract stimulates dauer recovery [27]. NAD^+^ triggers dauer recovery [48], but other components of the “food signal” are not known. We identified polypeptides as a food-associated cue for starved larvae. Perception of polypeptide in the form of casein, BSA, or tryptone in starved L1 larvae caused an irreversible arrest phenotype indistinguishable from that caused by perception of bacteria. Perception of polypeptide also caused GFP::DAF-16 to translocate to the cytoplasm in starved larvae. Furthermore, polypeptide triggered recovery from dauer arrest, a non-feeding state, suggesting polypeptide is a component of the “food signal”. Our results suggest a critical role of polypeptide perception in governing postembryonic development of free-living nematodes.

**Fig. 6:**
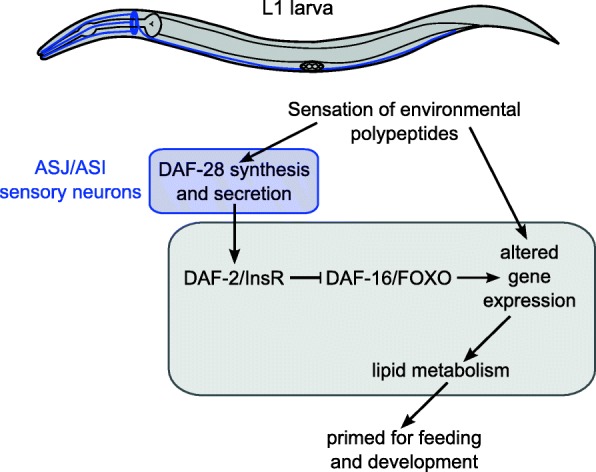
An organismal response to perception of food. Environmental polypeptides sensed by chemosensation leads to increased synthesis and secretion of the insulin-like peptide DAF-28, activating IIS. Additional, unknown pathways are also altered, which together with activated IIS affects gene expression and lipid metabolism. These physiological changes prime the animal for feeding and development, but they are deleterious if the animal does not actually feed

Together our results suggest a model in which chemosensation of environmental polypeptides promotes transcription and possibly secretion of DAF-28 from ASI and ASJ amphid neurons to mediate systemic effects on gene expression and metabolism (Fig. 6). These changes in gene expression and metabolism prime the animal for feeding and development, but they are deleterious in the absence of feeding and cause an irreversible developmental arrest. However, activation of IIS did not account for the entire gene expression response to food perception, nor did it account for the irreversible arrest phenotype. We conclude that food perception affects additional signaling pathways and that these pathways collaborate with IIS to regulate gene expression and metabolism.

## Conclusions

We conclude that *C. elegans* larvae sense environmental food-associated cues such as polypeptides and that this perception affects signaling, gene regulation, and metabolism. We suspect similar physiological effects of food perception, potentially in response to environmental polypeptides, are conserved among nematodes and more broadly among invertebrates. Worms use chemosensation to find food, and food perception may also serve to prime starved larvae for feeding and development. Such priming is apparently detrimental if not accompanied by feeding within hours, but we believe such a scenario where food cues are present without food is unnatural. In contrast, dauer larvae represent a common situation where starved larvae rely on perception to regulate development and metabolism. We show that dauer larvae exit arrest and resume development in response to perception of environmental polypeptides. Starved non-dauer larvae are able to feed immediately upon encountering food, but perception of environmental cues could accelerate the organismal response by not requiring ingestion and assimilation of nutrients. With a fluctuating food supply and boom and bust population dynamics in free-living nematodes, we suspect metabolic priming via food perception contributes to fitness by accelerating recovery from developmental arrest.

## Methods

### *C. elegans* strains and ivermectin treatment

Strains were maintained on agar plates containing standard nematode growth media (NGM) seeded with *E. coli* OP50 at 20 °C. Small liquid cultures were used to arrest larval development (see below), and *E. coli* HB101 was used as food in these cultures. Standard genetic techniques were used to make combinations of alleles. The wild-type strain N2 (Bristol) and the following mutant and transgenic strains were used:

- CB1370 *daf-2(e1370)*
  CF1038 *daf-16(mu86)*
  LRB268 *avr-14(vu47); glc-3(ok321) avr-15(vu227) glc-1(pk54); dukIs9[Pmyo-2::avr-15, Pmyo-2::mCherry, Pajm-1::AJM-1::GFP]*
  LRB269 *avr-14(vu47); glc-3(ok321) avr-15(vu227) glc-1(pk54); dukIs10[Pmyo-2::avr-15, Pmyo-2::mCherry, Phlh-8::GFP]*
  JD369 *avr-14(vu47); glc-3(ok321) avr-15(vu227) glc-1(pk54)*
  NK1228 *daf-16(mu86); unc-119(ed4); qyIs288 [Pdaf-16::GFP::DAF-16, unc-119(+)]*
  LRB338 *daf-16(mu86); daf-28(tm2308); qyIs289[Pdaf-16::GFP::DAF-16, unc-119 (+)]*
  LRB339 *daf-16(mu86); ins-4, ins-5, ins-6(hpDF761); daf-28(tm2308); qyIs289[Pdaf-16::GFP::DAF-16, unc-119(+)]*
  LRB340 *daf-16(mu86); ins-4, ins-5, ins-6(hpDF761); qyIs289[Pdaf-16::GFP::DAF-16, unc-119(+)]*
  GR1455 *mgls40[Pdaf-28::GFP]*

*dukIs9* injection mix contained the following: 1 ng/μL pCFJ90 (*Pmyo-2::mCherry*), 1 ng/μL pPD30_69_TK414_4A (*Pmyo-2::avr-15*), and 50 ng/μL pJS191 (*Pajm-1::AJM-1::GFP*). *dukIs10* injection mix contained the following: 1 ng/μL pCFJ90 (*Pmyo-2::mCherry*), 1 ng/μL pPD30_69_TK414_4A (*Pmyo-2::avr-15*), and 50 ng/μL pJKL464 (*Phlh-8::GFP*).

We used LRB268 and LRB269 for our primary analyses. These strains are sensitive to ivermectin in the pharynx alone, providing a controlled way to manipulate pharyngeal pumping. However, these strains have complex genetics, with four chromosomal mutations and a transgenic extrachromosomal array. In addition, the array carries the pharynx-specific *avr-15* transgene as well as a reporter gene. Consequently, these two strains were not amenable to analysis of other mutations and reporter genes. We determined that wild-type (N2) worms display similar responses to ivermectin and food (e.g., Fig. 3i), albeit at slightly different doses (see below). We proceeded to use the wild-type background for subsequent genetic analyses. The strain used is indicated for each figure or figure panel.

Ivermectin (Sigma) dissolved in DMSO was added to the appropriate cultures immediately following culture setup. Ivermectin dose was adjusted for different levels of resistance in different strains. We performed a dose response to ivermectin in each background and used the minimal effective dose that made each strain not eat, as assessed by growth (data not shown). LRB269 was treated with 20 ng/mL ivermectin, or 22.85 nM. LRB268 was treated with 50 ng/mL ivermectin. Strains in the N2 background, rather than the quadruple mutant background, were treated with 10 ng/mL. DMSO was added in equal amounts to control tubes. DMSO concentration ranged from 0.05 to 0.2%.

### Hypochlorite treatment and L1 arrest assays

Mixed-stage cultures of worms on 10-cm NGM plates were washed from the plates using virgin S-basal (S-basal lacking ethanol and cholesterol) and centrifuged for 1 min at 3000 rpm in 15-mL conical tubes. A hypochlorite solution (7:2:1 ddH_2_O, sodium hypochlorite (Sigma), 5 M KOH) was added to dissolve the animals. Worms were centrifuged after 1.5–2 min in the hypochlorite solution, and fresh solution was added. Total time in the hypochlorite solution was 7–10 min. Embryos were washed three times in virgin S-basal buffer (no ethanol or cholesterol) before final suspension in 3–6 mL virgin S-basal at a density of 1 worm/μL. Embryos were cultured in a 16-mm glass tube on a tissue culture roller drum at approximately 25 rpm and 21–22 °C.

For the M-cell division assay, 1 day following the hypochlorite treatment above, the worms were put in the appropriate condition (LB, tryptone, etc.) and cultured for 7 days before 100 larvae per replicate were examined on a Noble agar slide on a compound fluorescent microscope. For the seam cell division assay, 1 day following the hypochlorite treatment above, HB101 was added at 25 mg/mL for 2 days and the V1–6 cells on one side of the animal were scored for 60 larvae per replicate.

### Starvation recovery

Animals were treated in hypochlorite solution and suspended in virgin S-basal with DMSO or ivermectin as described above. Twenty-four hours after hypochlorite treatment, the appropriate bacteria (HB101 unless otherwise stated) or potential food cue was added at the appropriate dose (25 mg/mL for bacteria unless otherwise stated). HB101 filtrate was created by filtering HB101 at 25 mg/mL through a 22-μm filter. Yeast extract was at 5 mg/mL. Tryptone and casamino acids were at 10 mg/mL. Due to solubility limitations, casein and BSA were at 1 mg/mL. Ethanol was at 0.095% (*v*/*v*). Glucose was at 5% (*w*/*v*) or 278 mM. Amino acid solution (16 mg/mL) was prepared as in [25]. Addition of bacteria or potential food cues was considered the 0 h exposure time point (vertical dashed line in Fig. 1a). One hundred-microliter aliquots were sampled at the stated times up to 24 h and placed around the edge of a HB101 lawn on NGM plates. Number of plated worms (*T*_p_) was counted and the plates were incubated at 20 °C. After 3 days, the number of animals that recovered to at least the L4 stage (*T*_R_) was counted. Recovery was calculated as *T*_R_/*T*_p_.

### Fluorescent bead ingestion

Cultures were setup as for a starvation recovery experiment as above; except instead of plating the worms after 24 h, fluorescent beads (Fluoresbrite® YG Carboxylate Microspheres 0.10 μm from Polysciences) were added at 1:200 to the cultures. After 3–4 h, the cultures were examined on a Noble agar slide on a compound fluorescent microscope. The location of the fluorescent beads (i.e., gut, pharynx, or none inside the worm) was scored for 40 worms per replicate.

### α-Amanitin and cycloheximide treatment

Dose response curves with α-amanitin (Sigma) and cycloheximide (Sigma) were done using the *gpIs1* [P*hsp-16.2*::GFP] reporter [43] in a wild-type background to find a dose that prevented fluorescence in response to heat shock at 33 °C for 2 h (data not shown). These doses were determined to be 5 mM for cycloheximide and 25 μg/mL for α-amanitin. Both drug stocks were dissolved in water. The starvation recovery assay (Fig. 1d, e) was set up as above in the quadruple transgenic mutant, with drugs added 2 h before food addition and cultures washed three times with 10 mL virgin S-basal before plating.

### mRNA-seq and associated analysis

Worm cultures for LRB269 were set up using the hypochlorite treatment as described above, except in S-complete rather than virgin S-basal and scaled up to 20 mL per condition. Either ivermectin was added at 5 ng/mL or DMSO was added at 0.1%. Twenty-four hours after hypochlorite treatment, allowing for hatching and synchronization, HB101 was added at 25 mg/mL to the food tubes. Samples were collected at 1 h and 24 h after food addition. To collect the samples, worms were washed three times with 10 mL virgin S-basal then concentrated in 100 μL and frozen in liquid nitrogen. RNA was extracted with Trizol and chloroform. Libraries were prepared for sequencing using the NEBNext Ultra RNA Library Prep Kit for Illumina (E7530) with 250–400 ng of starting RNA per library and 13 cycles of PCR. Libraries were sequenced using Illumina HiSeq 4000. Bowtie was used to map reads to the WS210 genome [38]. Transcripts annotated in WS220 that were mapped to the WS210 genome coordinates were also included, as described previously [44]. Mapping efficiencies ranged from 78 to 85% for all libraries. HTSeq was used to generate count tables for each library [2]. Count tables were analyzed for differential expression using the edgeR package in R [56]. Detected genes were considered those expressed at a level of at least 1 count-per-million (CPM) in at least four libraries, reducing the number of genes included in the analysis to 18,190. The “calcNormFactors” function was used to normalize for RNA composition, and the tagwise dispersion estimate was used for differential expression analysis. The exact test was used for pairwise comparisons of conditions. Differentially expressed genes were considered those with an FDR < 0.05 and with |log_2_ (fold change)| > 0.5. Principal component analysis was performed using all libraries and all genes used in differential expression analysis (18,190 genes). Counts-per-million (CPM) values for each gene were mean-normalized across all libraries and log2 transformed prior to using the prcomp function in R. GO term analysis was performed using GOrilla [22, 23]. AmiGO 2 was accessed to download the genes in the metabolic process GO term (GO:0044710) and the larval development GO term (GO:0002164) [4, 13, 62]. GEO accession number for the dataset is GSE114955.

### GFP::DAF-16 localization

The *qyIs288 [Pdaf-16::GFP::DAF-16, unc-119(+)]* and qyIs289 *[Pdaf-16::GFP::DAF-16, unc-119(+)]* reporters [35] were analyzed in a *daf-16(mu86); unc-119(ed4)* mutant background. Standard genetic methods were used to cross *daf-28(tm2308)* and *ins-4, 5, 6(hpDf761)* into this background as well. Cultures were set up using the hypochlorite treatment as described above. One day later, HB101 was added at 25 mg/mL. One hour or 24 h after food addition, 50 larvae per replicate were examined on a Noble agar slide on a compound fluorescent microscope. For blocking translation, the assay was performed as described, except 5 mM cycloheximide was added 2 h before food addition.

### Reporter gene analysis

The *mgIs40* [P*daf-28*::GFP] reporter [40] was analyzed in a wild-type genetic background. Strain was maintained on NGM agar plates with *E. coli* OP50 as food at 20 °C. Eggs were prepared by standard hypochlorite treatment as described above. These eggs were used to set up a liquid culture consisting of virgin S-basal with a defined density of 1 worm/μL. Ivermectin was added at 10 ng/mL to the appropriate cultures. After 18 h to allow for hatching, the *E. coli* HB101 was added at 25 mg/mL to the fed samples. Six hours post food addition, the samples were washed three times with 10 mL S-basal and then run through the COPAS BioSorter measuring GFP fluorescence. Analysis of the COPAS data was performed in R. Data points were removed if they were determined to be debris by size. Fluorescence signal was normalized by optical extinction. For imaging, the samples were prepared in the same way then paralyzed with 3.75 mM sodium azide and placed on a Noble agar slide. Images were taken on a compound fluorescent microscope.

### Fixation and Nile red staining

Cultures of N2 wild-type were set up as for a starvation recovery experiment as above; except instead of plating the worms after 24 h, the cultures were washed three times with 10 mL virgin S-basal. Worms were concentrated in approximately 100 μL and frozen at − 80 °C. Fixation and staining protocol was modified from [52], using 1.7-mL Eppendorf tubes instead of 96-well plates and 200 μL solution additions instead of 150 μL. Images were taken on a compound fluorescent microscope. Fat droplets were quantified using the Analyze Particles function in ImageJ. Images were thresholded using negative controls to remove background. Minimum particle size was set as 1.3 mm^2^.

### Starvation survival

N2 wild-type animals were treated in hypochlorite solution and suspended in virgin S-basal or the appropriate media as described above. One hundred-microliter aliquots were sampled on different days and placed around the edge of an OP50 lawn on NGM plates. Number of plated worms (*T*_p_) was counted, and the plates were incubated at 20 °C. After 2 days, the number of animals that survived (*T*_s_) was counted. Survival was calculated as *T*_s_/*T*_p_. Survival curves were obtained by fitting survival data for each trial with the function

$$ S=100-\frac{100}{1+{e}^{\left({t}_{\mathrm{half}}-t\right)/\mathrm{rate}}} $$

### Quantitative image analysis of size

N2 worms were treated in hypochlorite solution and suspended in virgin S-basal or the appropriate media as described above. At the 50% survival times determined from starvation survival experiments, the worms were spun down at 3000 rpm for 1 min and pellets were transferred to OP50 seeded 10-cm NGM plates. Worms were allowed to recover for 48 h at 20 °C. Worms were then imaged and images were processed using the WormSizer plug-in for Fiji/ImageJ as described [46].

### Dauer recovery

N2 worms were treated in hypochlorite solution as described above then resuspended in S-complete at a density of 5 worms/μL and 1 mg/mL HB101 in 25-mL Erlenmeyer flasks [10]. Flasks were placed on a shaker at 20 °C for 7 days to form dauers. Cultures were spun down at 3000 rpm for 1 min. Supernatant was aspirated, and the appropriate media (LB, tryptone, etc.) was added, retaining a density of 5 worms/μL. Cultures were returned to shaker for 3 days. Approximately 75–100 worms were placed on a depression slide and scored as dauer, L4, or adult for each measurement.

### Data analysis and statistics

Data were handled in R and Excel. Graphs were plotted in the R packages ggplot2 or Vennerable or Excel. Statistical tests were performed in R or Excel. Starvation survival analysis was performed on 50% survival times (*t*_half_), which were obtained as in [35], with unpaired *t* tests performed where *n* is the number of replicates.

## Additional files

Additional file 1: **Figure S1.** Further characterization of ivermectin system and starvation recovery. (A) The proportion of larvae that displayed the stated localization of fluorescent beads three to four hours after bead addition is plotted for three biological replicates. (B-C) The proportion of larvae that recovered to at least the L4 stage after 3 days of recovery is plotted for three to four biological replicates. Ivermectin Resistant = JD369. Ivermectin Sensitive = LRB269. ***p < 0.001, *p < 0.05; unpaired *t* test. (A-C) Error bars are SEM. Quadruple mutant transgenic background. (PDF 32 kb)

Additional file 2: **Figure S2.** Ivermectin affects transcription in larvae. PCA of four biological replicates is plotted. Ellipses represent 80% confidence interval. Quadruple mutant transgenic background. (PDF 31 kb)

Additional file 3: Dataset S1. mRNA-seq analysis of food perception. Results of mRNA-seq analysis, including counts per gene, logFC, FDR, GOrilla analysis, and GO term gene lists used are included. (XLSX 18186 kb)

Additional file 4: **Figure S3.** GFP::DAF-16 localization responds to perception of many bacterial foods and requires *daf-2*. (A-B) GFP::DAF-16 localization is plotted for three to four biological replicates. ***p < 0.001; unpaired *t* test. Error bars are SEM. Wild-type background. (PDF 50 kb)

Additional file 5: **Table S1.** Feeding and food perception affect metabolism-related GO terms. Top fifteen GO terms ranked by FDR are listed for genes significant in IvF vs. IvS at 1 h and F vs. S at 1 h from four biological replicates of mRNA-seq. A quadruple mutant transgenic strain was used for mRNA-seq. Full list available in Additional file 3: Dataset S1. Table S2. Feeding but not food perception affects development-related GO terms. Top fifteen GO terms ranked by FDR are listed for genes significant in F vs. S at 1 h but not IvF vs. IvS at 1 h from four biological replicates of mRNA-seq. A quadruple mutant transgenic strain was used for mRNA-seq. Full list available in Additional file 3: Dataset S1. (DOCX 23 kb)

Additional file 6: **Figure S4.** Perception and physiological effects of potential food cues. (A) The proportion of larvae that recovered to at least the L4 stage after 3 days of recovery is plotted for three biological replicates. (B-C) GFP::DAF-16 localization is plotted for three to six biological replicates. (D) L1 starvation survival is plotted over time. A logistic regression of mean survival from three biological replicates is shown. (E) Worm length following 48 h of recovery is plotted relative to L1 starvation survival. (F) Worm length following 48 h of recovery is plotted as a density plot, showing altered population composition. (A-E) ***p < 0.001, **p < 0.01, *p < 0.05; unpaired *t* test. Error bars are SEM, except for in E where they are standard deviation. (A) Quadruple mutant transgenic background. (B-F) Wild-type background. (PDF 93 kb)
