# Supplementary figures and images for: Food perception without ingestion leads to metabolic changes and irreversible developmental arrest in C. elegans
Source: BMC Biol. 2018 Oct 8;16:112. doi: 10.1186/s12915-018-0579-3 (PMC6176503; doi:10.1186/s12915-018-0579-3)

Supp. Figure 1

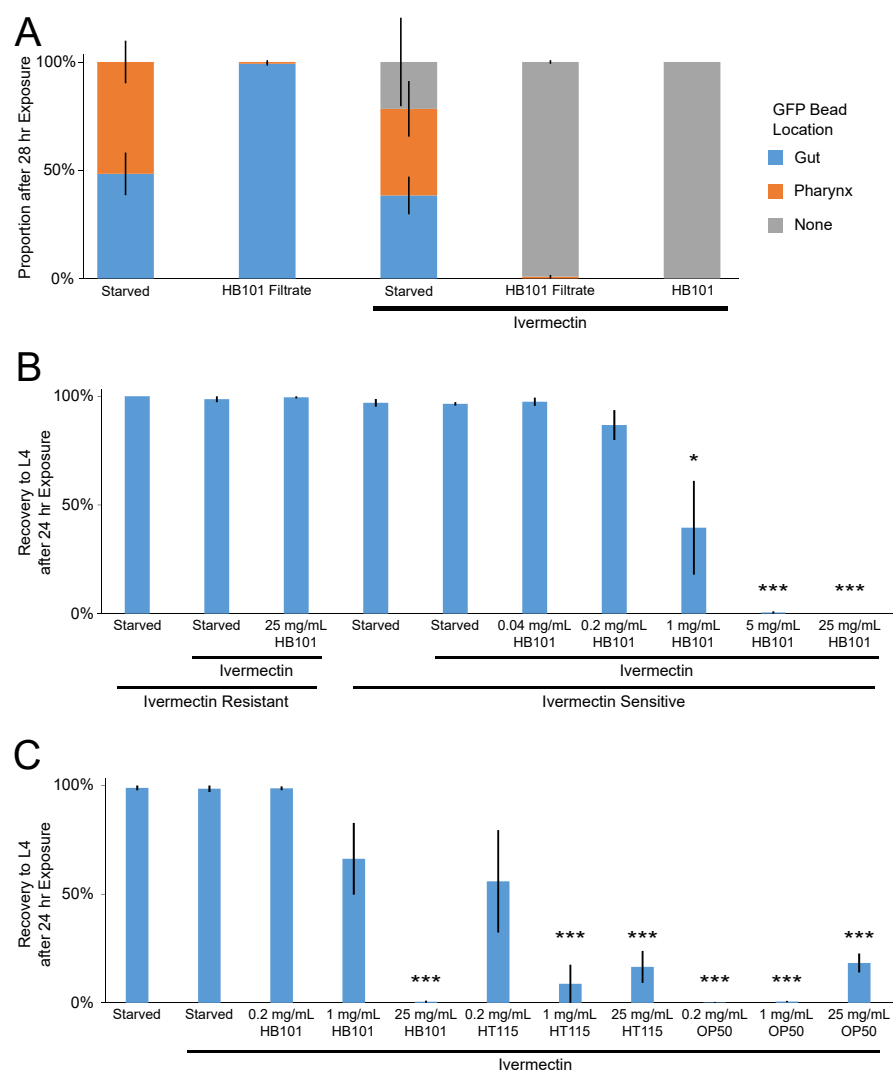

Supplement: Supplementary file 1 — Figure S1. Further characterization of ivermectin system and starvation recovery. (A) The proportion of larvae that displayed the stated localization of fluorescent beads three to four hours after bead addition is plotted for three biological replicates. (B-C) The proportion of larvae that recovered to at least the L4 stage after 3 days of recovery is plotted for three to four biological replicates. Ivermectin Resistant = JD369. Ivermectin Sensitive = LRB269. ***p < 0.001, *p < 0.05; unpaired t test. (A-C) Error bars are SEM. Quadruple mutant transgenic background. (PDF 32 kb) [file 12915_2018_579_MOESM1_ESM.pdf]

Supp. Figure 2

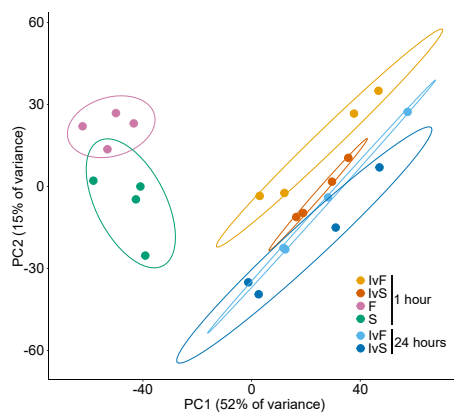

Supplement: Supplementary file 2 — Figure S2. Ivermectin affects transcription in larvae. PCA of four biological replicates is plotted. Ellipses represent 80% confidence interval. Quadruple mutant transgenic background. (PDF 31 kb) [file 12915_2018_579_MOESM2_ESM.pdf]

Supp. Figure 3

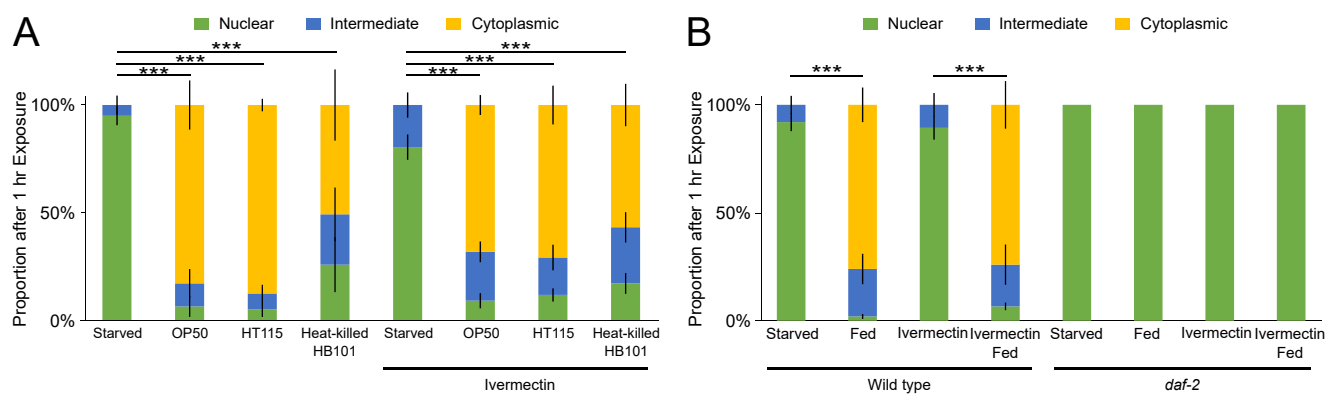

Supplement: Supplementary file 4 — Figure S3. GFP::DAF-16 localization responds to perception of many bacterial foods and requires daf-2. (A-B) GFP::DAF-16 localization is plotted for three to four biological replicates. ***p < 0.001; unpaired t test. Error bars are SEM. Wild-type background. (PDF 50 kb) [file 12915_2018_579_MOESM4_ESM.pdf]

Supp. Figure 4

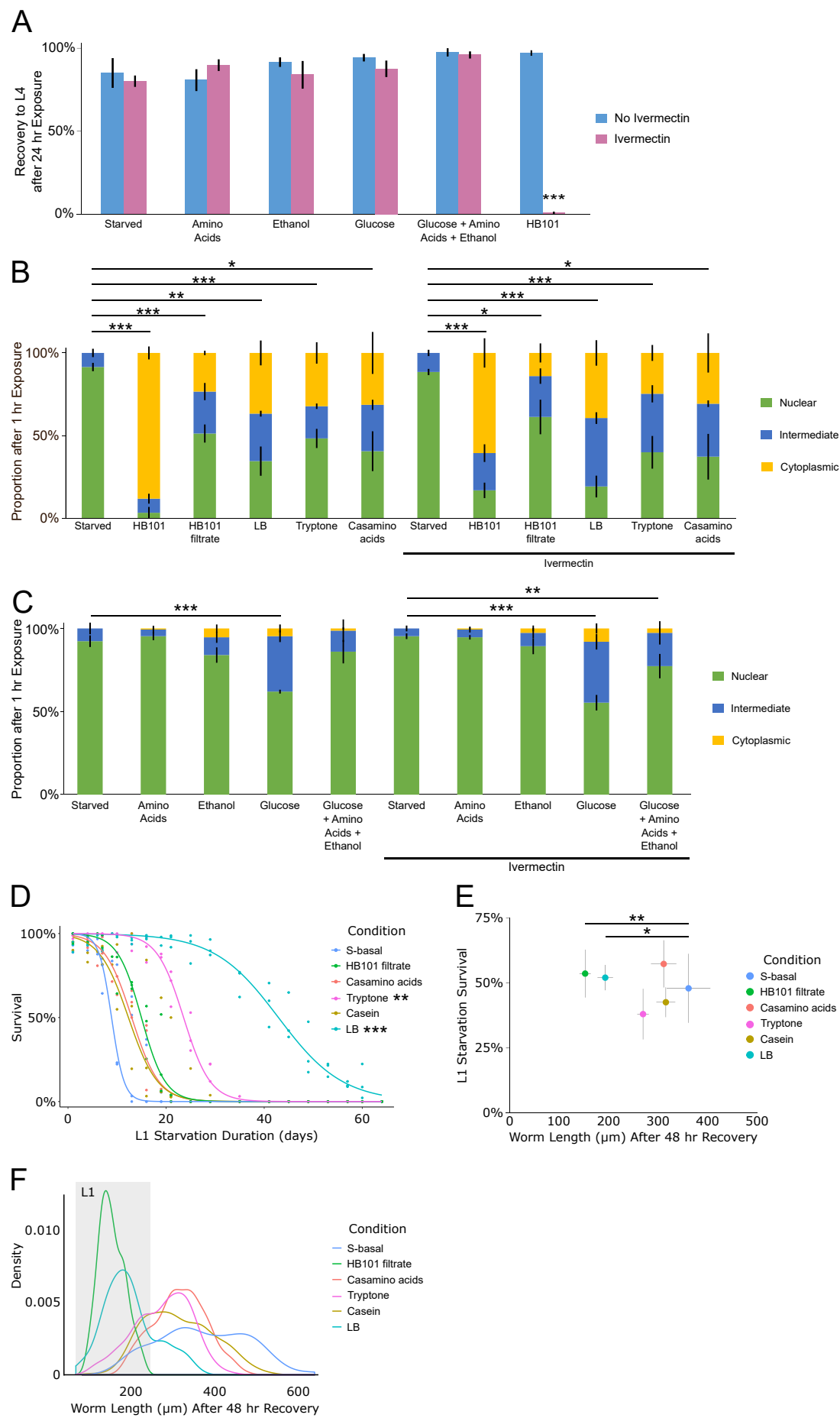

Supplement: Supplementary file 6 — Figure S4. Perception and physiological effects of potential food cues. (A) The proportion of larvae that recovered to at least the L4 stage after 3 days of recovery is plotted for three biological replicates. (B-C) GFP::DAF-16 localization is plotted for three to six biological replicates. (D) L1 starvation survival is plotted over time. A logistic regression of mean survival from three biological replicates is shown. (E) Worm length following 48 h of recovery is plotted relative to L1 starvation survival. (F) Worm length following 48 h of recovery is plotted as a density plot, showing altered population composition. (A-E) ***p < 0.001, **p < 0.01, *p < 0.05; unpaired t test. Error bars are SEM, except for in E where they are standard deviation. (A) Quadruple mutant transgenic background. (B-F) Wild-type background. (PDF 93 kb) [file 12915_2018_579_MOESM6_ESM.pdf]
