# Supplementary material for: Food perception without ingestion leads to metabolic changes and irreversible developmental arrest in C. elegans
Source: BMC Biol. 2018 Oct 8;16:112. doi: 10.1186/s12915-018-0579-3 (PMC6176503; doi:10.1186/s12915-018-0579-3)
Supplement: Supplementary file 5 — Table S1. Feeding and food perception affect metabolism-related GO terms. Top fifteen GO terms ranked by FDR are listed for genes significant in IvF vs. IvS at 1 h and F vs. S at 1 h from four biological replicates of mRNA-seq. A quadruple mutant transgenic strain was used for mRNA-seq. Full list available in Additional file 3: Dataset S1. Table S2. Feeding but not food perception affects development-related GO terms. Top fifteen GO terms ranked by FDR are listed for genes significant in F vs. S at 1 h but not IvF vs. IvS at 1 h from four biological replicates of mRNA-seq. A quadruple mutant transgenic strain was used for mRNA-seq. Full list available in Additional file 3: Dataset S1. (DOCX 23 kb) [file 12915_2018_579_MOESM5_ESM.docx]

Additional file 5: **Table S1**. Feeding and food perception affect metabolism-related GO terms.

| GO term Description | FDR | Enrichment | Number of genes in GO term | Number of genes in target set | Number of genes in overlap |
| --- | --- | --- | --- | --- | --- |
| Single-organism metabolic process | 3.44E-25 | 2.24 | 1587 | 737 | 192 |
| Monocarboxylic acid metabolic process | 6.49E-20 | 4.82 | 211 | 737 | 55 |
| Flavonoid biosynthetic process | 6.93E-20 | 8.58 | 69 | 737 | 32 |
| Flavonoid metabolic process | 7.91E-20 | 8.58 | 69 | 737 | 32 |
| Carboxylic acid metabolic process | 8.59E-20 | 3.56 | 390 | 737 | 75 |
| Glucuronate metabolic process | 9.23E-20 | 8.58 | 69 | 737 | 32 |
| Uronic acid metabolic process | 1.11E-19 | 8.58 | 69 | 737 | 32 |
| Cellular glucuronidation | 1.39E-19 | 8.58 | 69 | 737 | 32 |
| Flavonoid glucuronidation | 1.85E-19 | 8.58 | 69 | 737 | 32 |
| Organic acid metabolic process | 1.03E-18 | 3.41 | 407 | 737 | 75 |
| Oxoacid metabolic process | 1.13E-18 | 3.41 | 407 | 737 | 75 |
| Small molecule metabolic process | 2.21E-17 | 2.70 | 671 | 737 | 98 |
| Oxidation-reduction process | 4.27E-17 | 2.84 | 579 | 737 | 89 |
| Monosaccharide metabolic process | 6.32E-17 | 6.35 | 102 | 737 | 35 |
| Innate immune response | 2.66E-11 | 3.27 | 283 | 737 | 50 |

Additional Table 1. Top fifteen GO terms ranked by FDR are listed for genes significant in IvF vs. IvS at 1 hr and F vs. S at 1 hr from four biological replicates of mRNA-seq. A quadruple mutant transgenic strain was used for mRNA-seq. Full list available in S1 Dataset.

**Table S2**. Feeding but not food perception affects development-related GO terms.

| GO term Description | FDR | Enrichment | Number of genes in GO term | Number of genes in target set | Number of genes in overlap |
| --- | --- | --- | --- | --- | --- |
| Cellular nitrogen compound metabolic process | 4.60E-35 | 1.50 | 1884 | 3404 | 704 |
| Embryo development ending in birth or egg hatching | 5.74E-35 | 1.38 | 2889 | 3404 | 995 |
| Embryo development | 1.05E-34 | 1.38 | 2892 | 3404 | 996 |
| Nucleic acid metabolic process | 1.70E-34 | 1.60 | 1340 | 3404 | 536 |
| Nucleobase-containing compound metabolic process | 4.74E-33 | 1.54 | 1576 | 3404 | 604 |
| Reproduction | 5.74E-33 | 1.45 | 1576 | 3404 | 754 |
| Heterocycle metabolic process | 1.10E-32 | 1.52 | 1621 | 3404 | 616 |
| Cellular aromatic compound metabolic process | 9.27E-32 | 1.51 | 1625 | 3404 | 614 |
| Multicellular organism development | 1.75E-31 | 1.31 | 3520 | 3404 | 1153 |
| Organic cyclic compound metabolic process | 2.16E-31 | 1.50 | 1670 | 3404 | 626 |
| RNA metabolic process | 6.17E-29 | 1.61 | 1122 | 3404 | 450 |
| Anatomical structure development | 7.47E-29 | 1.28 | 3857 | 3404 | 1232 |
| Single organism reproductive process | 3.14E-28 | 1.53 | 1395 | 3404 | 532 |
| RNA processing | 2.67E-26 | 2.06 | 373 | 3404 | 192 |
| Single-multicellular organism process | 2.69E-26 | 1.26 | 4091 | 3404 | 1282 |

Additional Table 2. Top fifteen GO terms ranked by FDR are listed for genes significant in F vs. S at 1 hr but not IvF vs. IvS at 1 hr from four biological replicates of mRNA-seq. A quadruple mutant transgenic strain was used for mRNA-seq. Full list available in S1 Dataset.
